# Supplementary figures and images for: RADA-dependent branch migration has a predominant role in plant mitochondria and its defect leads to mtDNA instability and cell cycle arrest
Source: PLoS Genet. 2022 May 12;18(5):e1010202. doi: 10.1371/journal.pgen.1010202 (PMC9129000; doi:10.1371/journal.pgen.1010202)

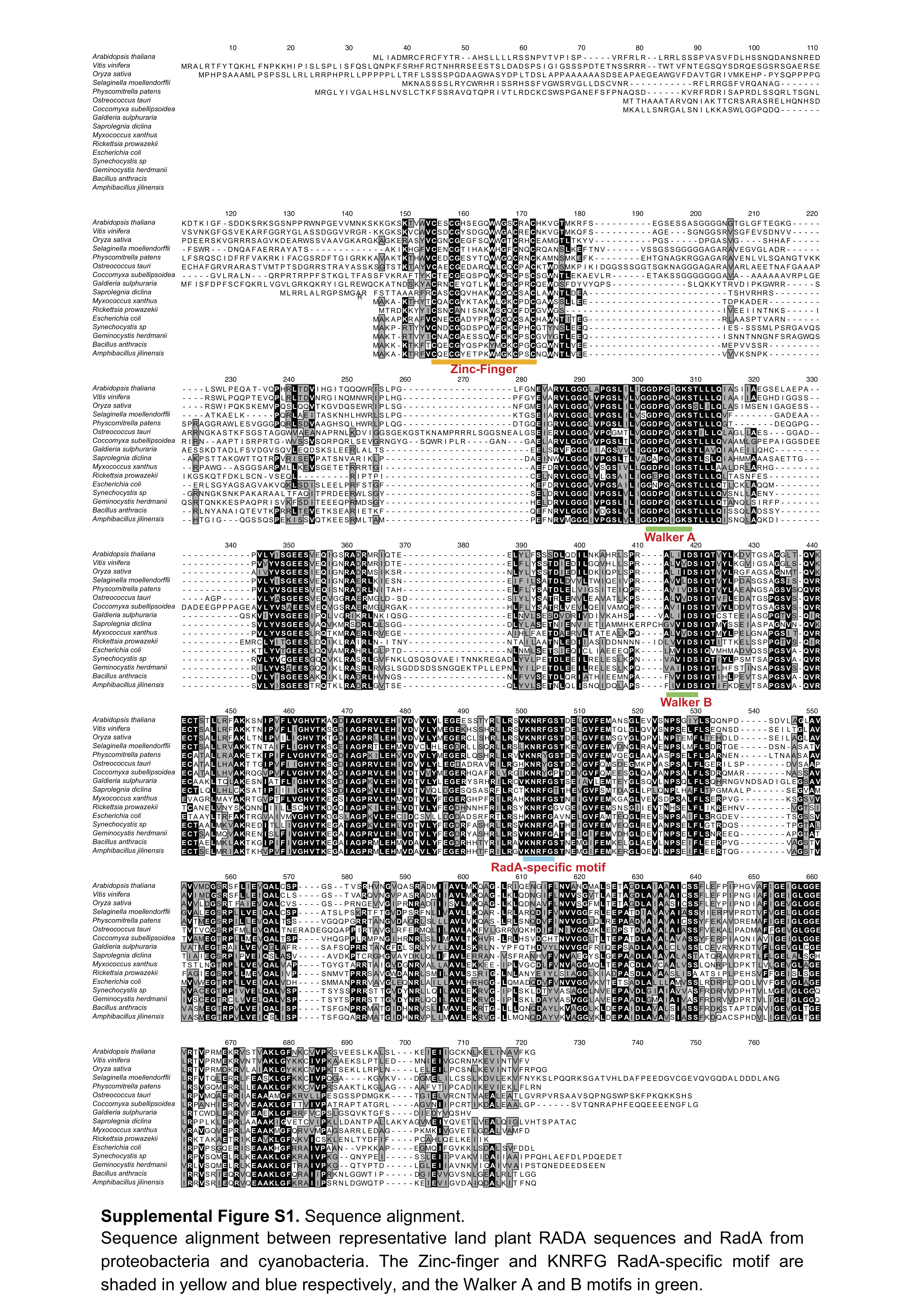

Supplement: S1 Fig — Sequence alignment between representative land plant RADA sequences and RadA from proteobacteria and cyanobacteria. The Zinc-finger and KNRFG RadA-specific motif are shaded in yellow and blue respectively, and the Walker A and B motifs in green. (TIFF) [file pgen.1010202.s001.tiff]

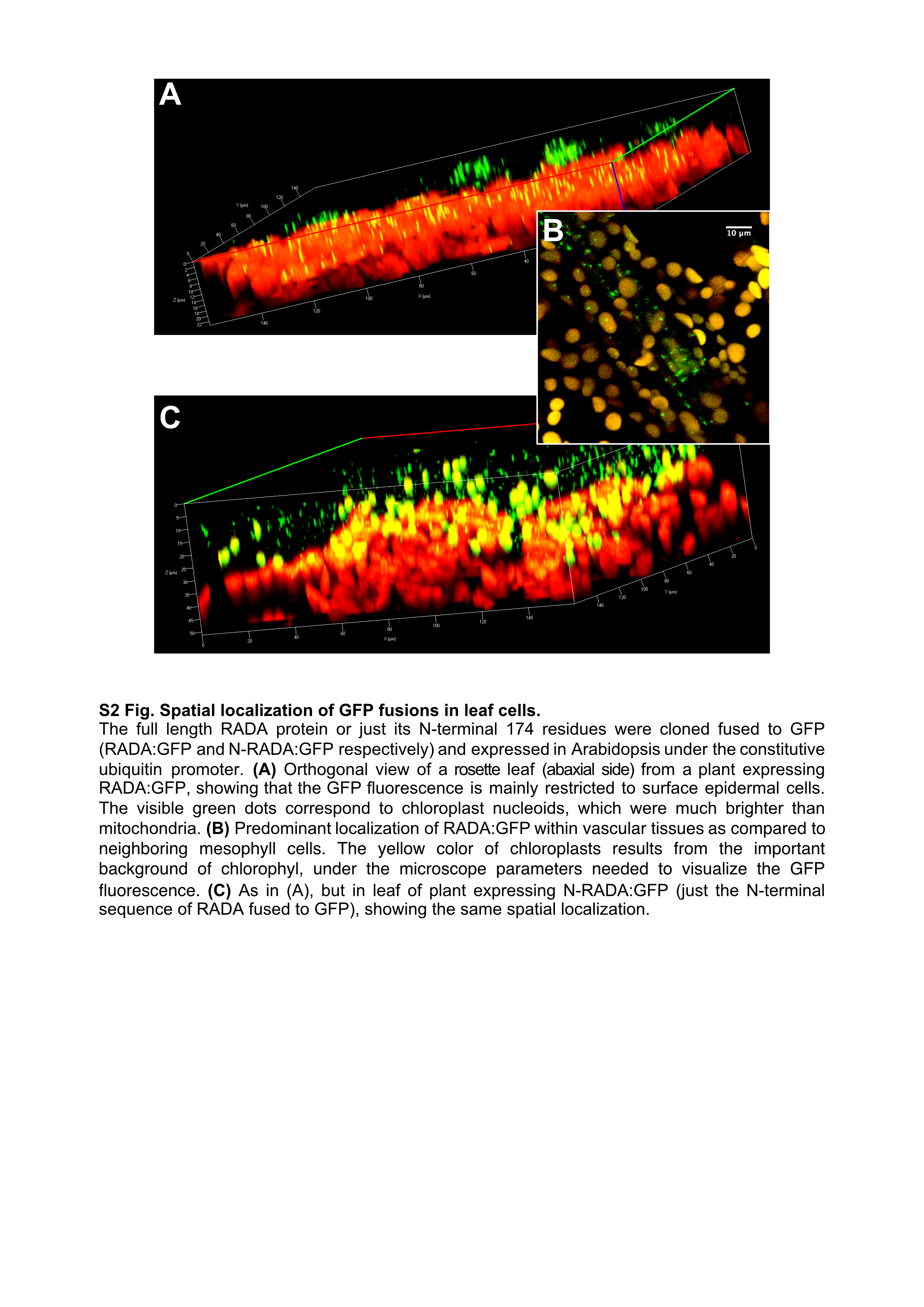

Supplement: S2 Fig — The full length RADA protein or just its N-terminal 174 residues were cloned fused to GFP (RADA:GFP and N-RADA:GFP respectively) and expressed in Arabidopsis under the constitutive ubiquitin promoter. (A) Orthogonal view of a rosette leaf (abaxial side) from a plant expressing RADA:GFP, showing that the GFP fluorescence is mainly restricted to surface epidermal cells. The visible green dots correspond to chloroplast nucleoids, which were much brighter than mitochondria. (B) Predominant localization of RADA:GFP within vascular tissues as compared to neighboring mesophyll cells. The yellow color of chloroplasts results from the important background of chlorophyl, under the microscope parameters needed to visualize the GFP fluorescence. (C) As in (A), but in leaf of plant expressing N-RADA:GFP (just the N-terminal sequence of RADA fused to GFP), showing the same spatial localization. (TIFF) [file pgen.1010202.s002.tiff]

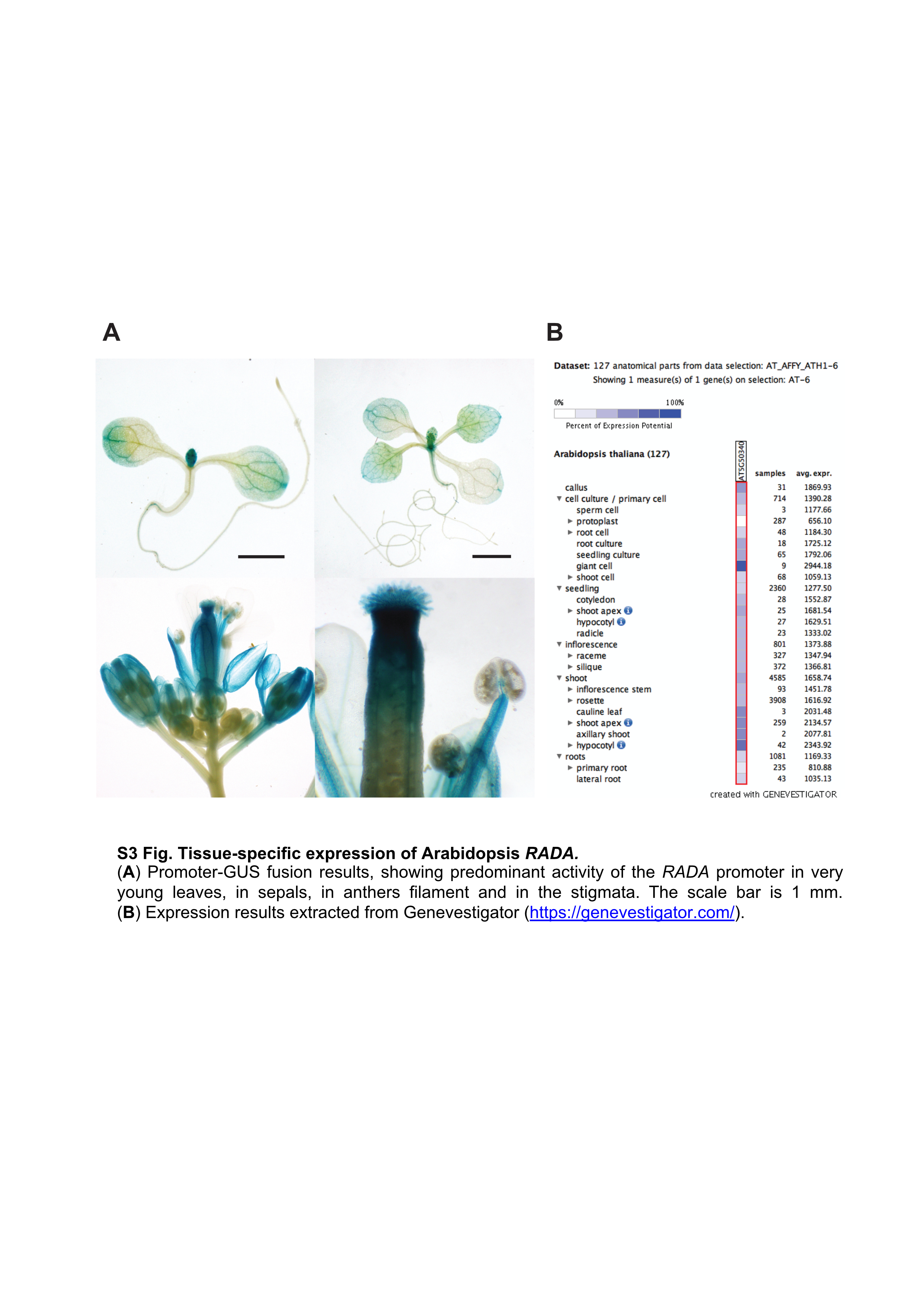

Supplement: S3 Fig — (A) Promoter-GUS fusion results, showing predominant activity of the RADA promoter in very young leaves, in sepals, in anthers filament and in the stigmata. The scale bar is 1 mm. (B) Expression results extracted from Genevestigator (https://genevestigator.com/). (TIFF) [file pgen.1010202.s003.tiff]

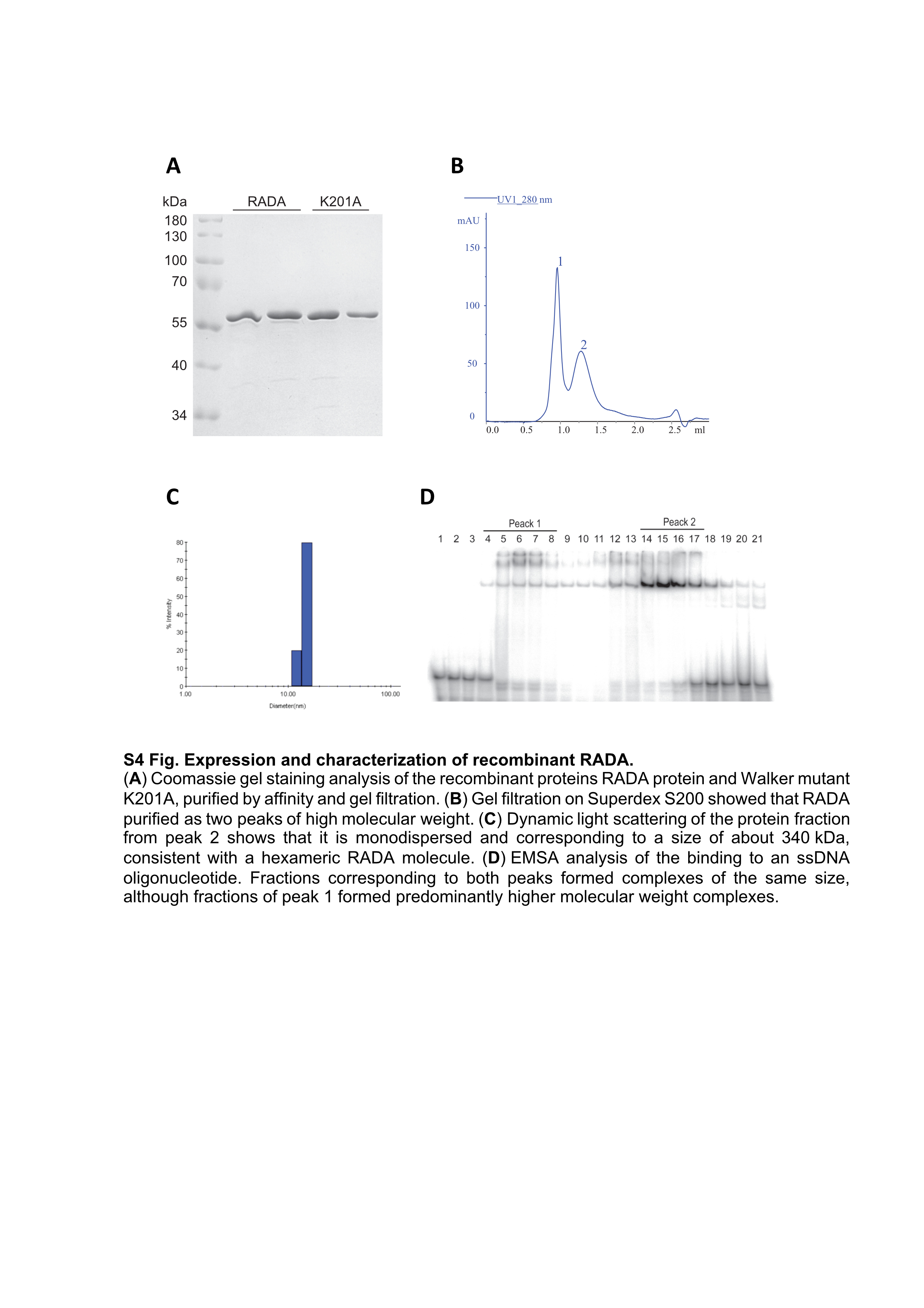

Supplement: S4 Fig — (A) Coomassie gel staining analysis of the recombinant proteins RADA protein and Walker mutant K201A, purified by affinity and gel filtration. (B) Gel filtration on Superdex S200 showed that RADA purified as two peaks of high molecular weight. (C) Dynamic light scattering of the protein fraction from peak 2 shows that it is monodispersed and corresponding to a size of about 340 kDa, consistent with a hexameric RADA molecule. (D) EMSA analysis of the binding to an ssDNA oligonucleotide. Fractions corresponding to both peaks formed complexes of the same size, although fractions of peak 1 formed predominantly higher molecular weight complexes. (TIFF) [file pgen.1010202.s004.tiff]

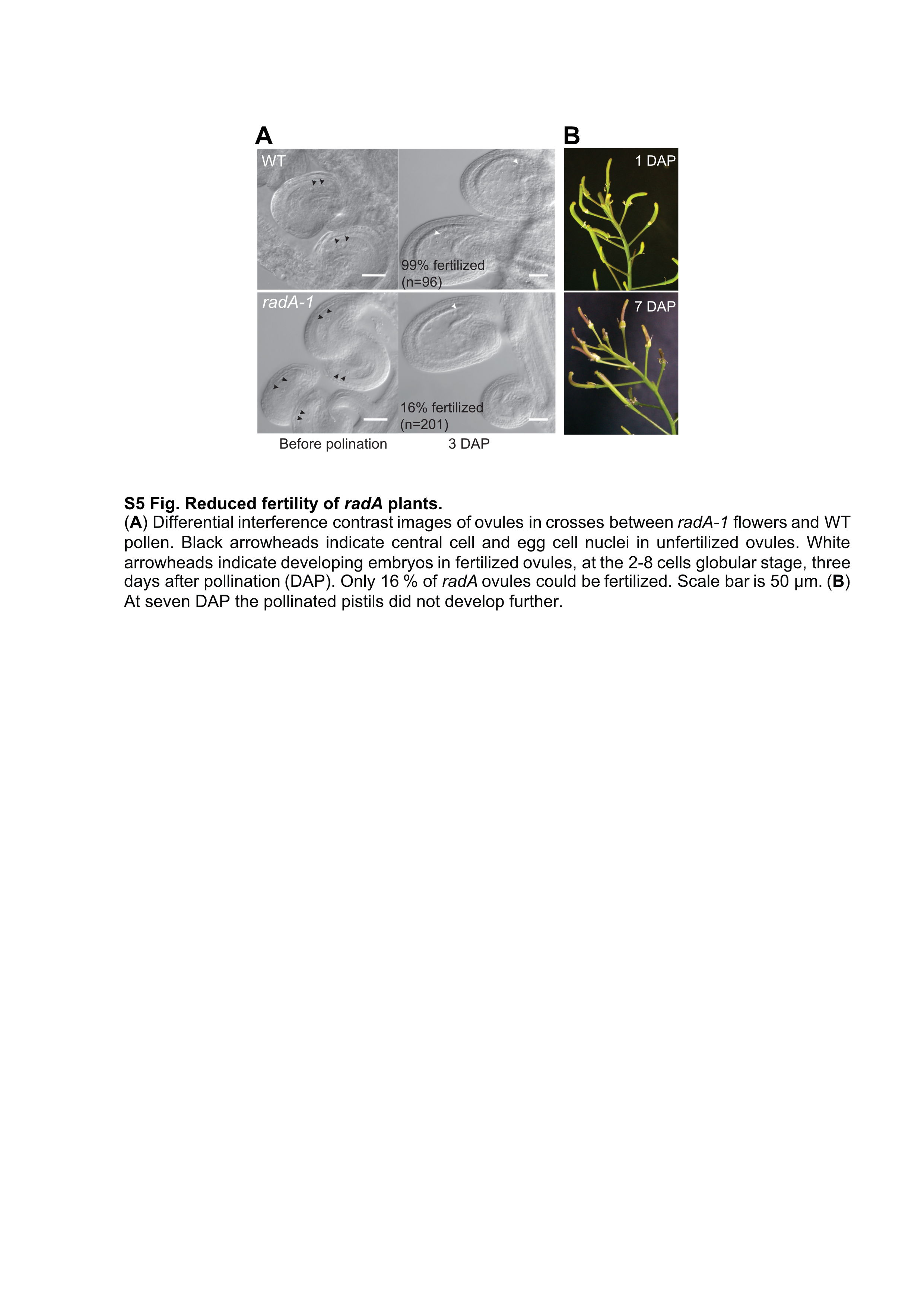

Supplement: S5 Fig — (A) Differential interference contrast images of ovules in crosses between radA-1 flowers and WT pollen. Black arrowheads indicate central cell and egg cell nuclei in unfertilized ovules. White arrowheads indicate developing embryos in fertilized ovules, at the 2–8 cells globular stage, three days after pollination (DAP). Only 16% of radA ovules could be fertilized. Scale bar is 50 μm. (B) At seven DAP the pollinated pistils did not develop further. (TIFF) [file pgen.1010202.s005.tiff]

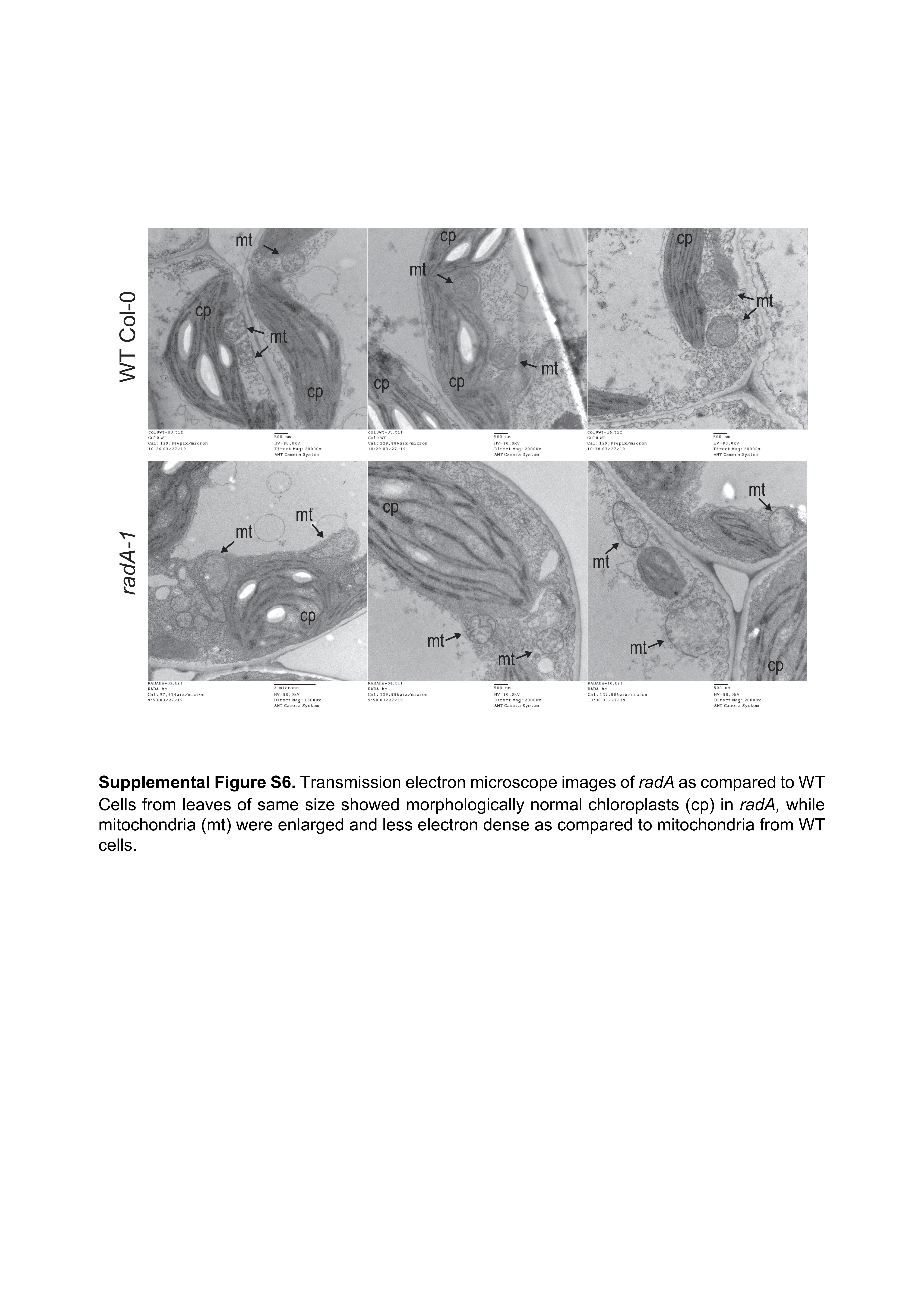

Supplement: S6 Fig — Cells from leaves of same size showed morphologically normal chloroplasts (cp) in radA, while mitochondria (mt) were enlarged and less electron dense as compared to mitochondria from WT cells. (TIFF) [file pgen.1010202.s006.tiff]

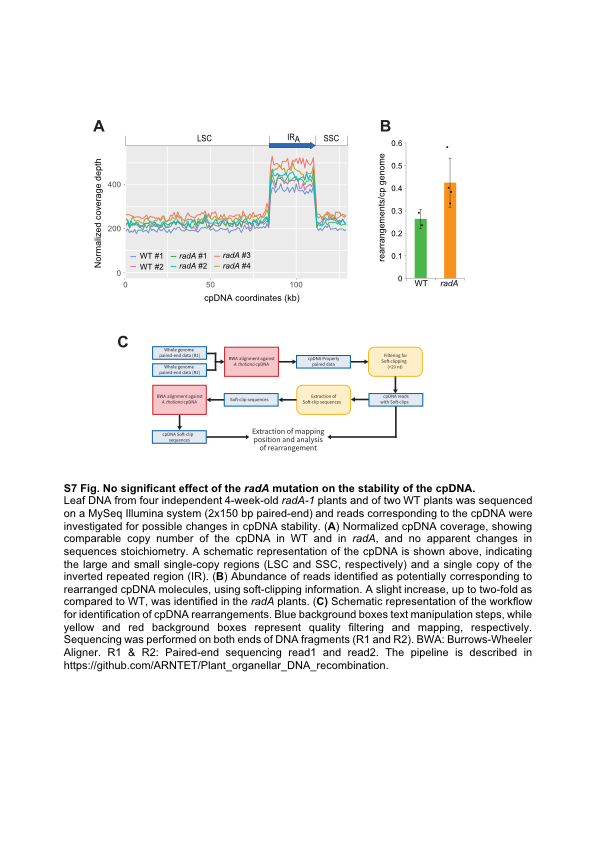

Supplement: S7 Fig — Leaf DNA from four independent 4-week-old radA-1 plants and of two WT plants was sequenced on a MySeq Illumina system (2x150 bp paired-end) and reads corresponding to the cpDNA were investigated for possible changes in cpDNA stability. (A) Normalized cpDNA coverage, showing comparable copy number of the cpDNA in WT and in radA, and no apparent changes in sequences stoichiometry. A schematic representation of the cpDNA is shown above, indicating the large and small single-copy regions (LSC and SSC, respectively) and a single copy of the inverted repeated region (IR). (B) Abundance of reads identified as potentially corresponding to rearranged cpDNA molecules, using soft-clipping information. A slight increase, up to two-fold as compared to WT, was identified in the radA plants. (C) Schematic representation of the workflow for identification of cpDNA rearrangements. Blue background boxes text manipulation steps, while yellow and red background boxes represent quality filtering and mapping, respectively. Sequencing was performed on both ends of DNA fragments (R1 and R2). BWA: Burrows-Wheeler Aligner. R1 & R2: Paired-end sequencing read1 and read2. The pipeline is described in https://github.com/ARNTET/Plant_organellar_DNA_recombination. (TIFF) [file pgen.1010202.s007.tiff]

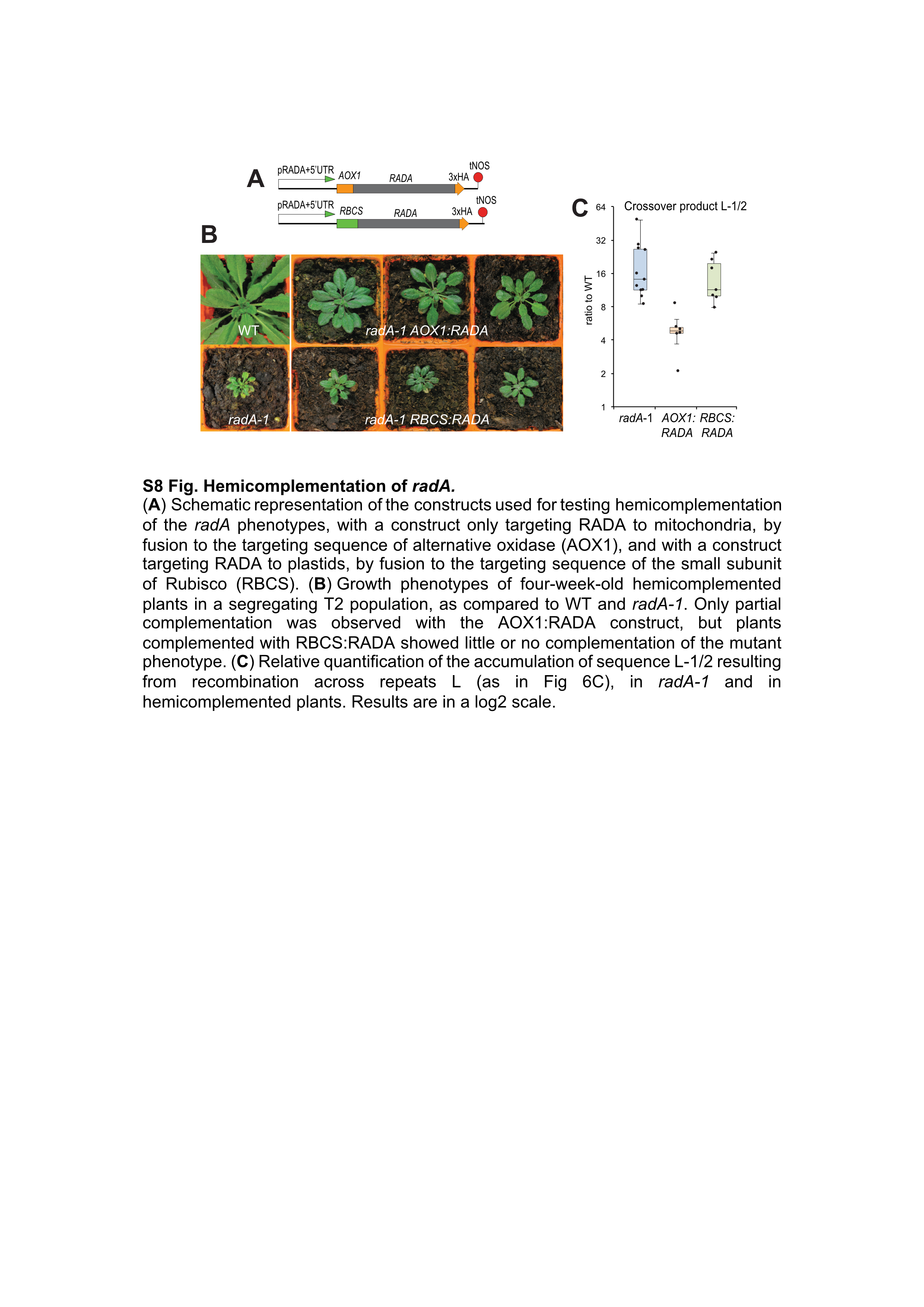

Supplement: S8 Fig — (A) Schematic representation of the constructs used for testing hemicomplementation of the radA phenotypes, with a construct only targeting RADA to mitochondria, by fusion to the targeting sequence of alternative oxidase (AOX1), and with a construct targeting RADA to plastids, by fusion to the targeting sequence of the small subunit of Rubisco (RBCS). (B) Growth phenotypes of four-week-old hemicomplemented plants in a segregating T2 population, as compared to WT and radA-1. Only partial complementation was observed with the AOX1:RADA construct, but plants complemented with RBCS:RADA showed little or no complementation of the mutant phenotype. (C) Relative quantification of the accumulation of sequence L-1/2 resulting from recombination across repeats L (as in Fig 6C), in radA-1 and in hemicomplemented plants. Results are in a log2 scale. (TIFF) [file pgen.1010202.s008.tiff]

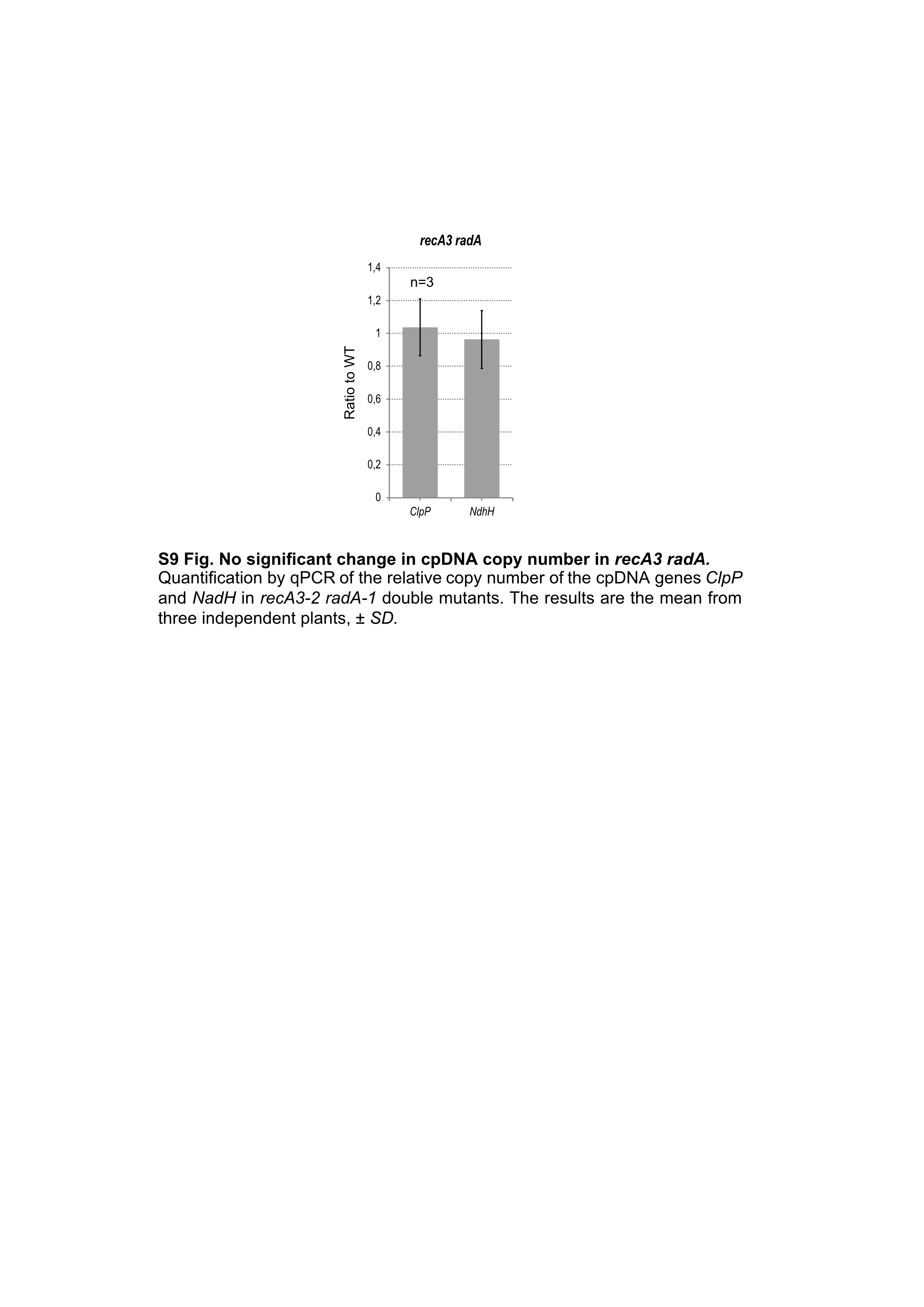

Supplement: S9 Fig — Quantification by qPCR of the relative copy number of the cpDNA genes ClpP and NadH in recA3-2 radA-1 double mutants. The results are the mean from three independent plants, ± SD. (TIFF) [file pgen.1010202.s009.tiff]
