# Supplementary material for: RADA-dependent branch migration has a predominant role in plant mitochondria and its defect leads to mtDNA instability and cell cycle arrest
Source: PLoS Genet. 2022 May 12;18(5):e1010202. doi: 10.1371/journal.pgen.1010202 (PMC9129000; doi:10.1371/journal.pgen.1010202)
Supplement: Attachment — Submitted filename: Response to Reviewers.docx [file pgen.1010202.s011.docx]

Response to Reviewers

First of all, we would like to thank the reviewers for their positive appreciation of our work and constructive comments. We have incorporated all corrections. Our answers and comments are indicated in blue

Reviewer #1:

Comments and suggestions for improvement:

Introduction:
In the first paragraph the involvement of different size repeats in recombination is introduced. However, this statement should be rephrased to be more accurate.
What is the evidence for illegitimate recombination of repeats of 100-500 bp in size in wild type plants (which is implied in the introduction)? These repeats could be involved homologous recombination as they have sufficient length of similar sequences, though at lower frequency than the large repeats.
If so, I would clarify that illegitimate recombination would occur at short repeats, instead of intermediate-size repeats.
Also, I understand that HR between large repeats is also considered ectopic. Please, clarify the use of ectopic when referring to IRs.

We agree with the reviewer that it is not easy to define in just a couple of words the concept of "infrequent recombination involving relatively small repeated sequences". Indeed, illegitimate recombination cannot be applied to homologous recombination involving repeated sequences that are several hundreds of nucleotides in size. Illegitimate can be applied to microhomology-mediated recombination. We often used the term "ectopic recombination", but we agree that ectopic applies to recombination involving any size of repeats. Terms such as "non-allelic recombination" that are often used in the literature were considered, but they are misleading in the context of the polyploid organellar genomes.

We have therefore, as possible, through the text replaced "ectopic recombination" by "recombination across repeated sequences", or by "recombination involving IRs". In the introduction we also explain that recombination involving IRs and microhomologies is normally asymmetric, because involving the BIR pathway and that only one of the two possible product is formed.

The first sentence of the second paragraph should be revised. “Defect of the” should probably be deleted.

Indeed. It was corrected

Results:

Figure 1A. Phaeophytes (now Phaeophyceae) are part of the monophyletic Stramenopiles. See Adl et al. 2019 doi:10.1111/jeu.12691

Thank you for the information. It is now corrected in the new Figure 1A

Fig1A legend: what is the meaning of the numbers on branches? and of the scale bar?

Numbers are bootstrap support (%, from 1000 iterations). Branches with values below 70% are collapsed. The scale bar indicates nucleotide substitutions per site. The information is now given in the Figure legend.

Also Materials and Methods:
Phylogenetic inference should be done using the high-performance methods based on ML and MP criteria, instead of NJ. The size of the alignment should be easily analyzed under ML with 100 to 1,000 bootstrap pseudoreplicates using PhyML or RaxML. Boosstrap support values should be shown on the branches of the best tree.

We have built a new maximum likelihood tree with IQ-Tree, as proposed by the editor, from 1000 bootstrap replications. The bootstrap values are indicated. The main conclusions remain unchanged.

Figure 2. In the legend, 2D is mentioned, which is not present and 2C is not mentioned. Theytype of tissue shown should be detailed in the legend.

Thank you for the correction. We have corrected the legend and specify that it corresponds to epidermal cells of the abaxial side of rosette leaves.

“We also found RADA:GFP mainly localized in the epidermis and vascular tissue of the Arabidopsis rosette leaves (Supplemental Figure S2A).”
Figure S2 does not show evidence for the localization in vascular tissue. Where is that evidence presented?
Figure S2 legend; clarify if these are rosette leaves. Is vascular tissue shown? Explain N-RADA:FGP versus RADA:GFP.

We now added an additional image (Figure S2B) showing preferential localization of RADA:GFP in vascular tissue as compared to neighboring mesophyll cells. As explained in the legend, the yellowish color of chloroplasts in the figure results from the important background of chlorophyl, under the microscope parameters needed to visualize the GFP fluorescence. In the modified legend we explain that RADA:GFP and N-RADA:GFP are the full protein or just the non-conserved N-terminal part of the protein fused to GFP, respectively. We also specify that the images are from the abaxial side of rosette leaves.

“However, our observations of RADA:GFP expressed in different plant tissues (leaves, roots
and flowers) did not give any hint that Arabidopsis RADA could be also targeted to the
nucleus.”
So far in the text, there is no evidence of the expression in different plant tissues. If you refer to the information in Fig. S3, indicate so or move to this statement to the following paragraph.

Indeed, the images were not presented. At the time it was unfortunately not documented. To grow plants up to flowering stage for new pictures will take some time, and because it is not of major relevance for our work, we prefer to no longer cite the observations in roots and flowers, and just state that "…our observations of RADA:GFP did not give any hint that Arabidopsis RADA is also targeted to the nucleus (Figures 2 and S2)".

Figure 4:Explain the meaning of the annotations in the x-axis in the legend of figure 4F.

The values correspond to the serial dilutions of the cell suspensions that were spotted on the agarose plates. It is now explained in the figure legend.

“We tested by qPCR the accumulation of crossover products for repeats F, L and EE and, as expected, in all plants there was a significant increase in crossover products versus WT levels (Figure 6C),”
I disagree with the use of the term “crossover products” as it implies that those alternative arrangements are the result of crossover. Instead, and as stated later, they could and likely are the result of BIR. Thus, I would change crossover products to "alternative arrangements or conformations.

We changed the text accordingly. As explained above, we have as possible utilized the terms "recombination across repeated sequences", or "recombination involving IRs", as well as "alternative conformations" as proposed by the reviewer.

“which could be because of alternative mtDNA repair by the error-prone break induced
replication (BIR) pathway [6, 7].”
None of the HR pathways are error free, although BIR is usually regarded as the most error-prone. The types of mutations observed in HR repair include base pair substitutions, indels, and complex chromosome rearrangements. Did you observe any? did you observe polymorphisms in the DNAseq reads of the regions resulting from BIR?

When using the term "error-prone" we referred to the propension of BIR to reinitiate replication at repeated sequence or microhomologies. We deleted the term, because it is indeed misleading and can be interpreted as an increase in SNP and indels.

Regarding the question of the reviewer, whether we observed polymorphisms in the DNAseq reads in the regions resulting from BIR, at the level of the repeated sequences. We did not find evidence for polymorphisms in the sequences of repeats L and F. However, in the sequence of small repeat EE we found that several reads shared the same base substitutions, as compared to the reference. Because it was not consistent and because we cannot be sure that such SNPs are not the result of sequence errors, we preferred not to discuss it. To differentiate sequence errors from a true increase in mutation rates we would need to use duplex sequencing, as described in Wu et al. 2020 (doi/10.1073/pnas.2001998117)

“. Hoever, these values are misleading because compared to the basal levels that exist in WT plants.”
Please fix the grammar to improve this sentence.

Done

Bioinformatic analysis:
Please include the parameters used for the alignment using BWA. Note that depending on the parameters, a read that aligns to two regions of the reference sequence (i.e. a repeat) could be aligned twice (to both regions) or only once (i.e. randomly to only one of the two copies of the repeat). thus, the resulting figure and interpretation would be quite different. Also, a range of mismatches may be allowed for alignment depending on the parameters provided. Thus, the parameters provide key information to understand the results.
Same for bowtie alignment.

The parameters are now given in the methods section.

It is not clear what do you mean by “short-clipping sequence”? Is it part of the Illumina read that does not align with the reference sequence? Which % of the read can it represent?

It was an error. It refers to "soft-clips". The soft clipping information was used as a proxy to evaluate the abundance of cpDNA structural variants involving just a few nucleotides. As described in the pipeline, we extracted reads that align to the cpDNA, at least 20 nucleotides, and that contain a soft-clipped regions that also aligns to the cpDNA. We manually eliminated those that map to the borders of the inverted repeats. The pipeline is described in more detail in <https://github.com/ARNTET/Plant_organellar_DNA_recombination>

Why is this section focused on the analysis of the cpDNA and not the mtDNA? I’m not sure if or why the mtDNA was analyzed differently.

In this section we were mainly focused in testing if RADA had any detectable effect on the stability of the chloroplast genome. Because we could not identify any changes in copy number or sequences stoichiometry, as observed for the mtDNA, we tried to identify an eventual increase in rearrangements involving no sequence homologies or microhomologies, as previously described for several mutants affected in cpDNA maintenance (*why1why3, why1why3recA1…*). We also tried a PCR-based approach using divergent primers, has described by others to detect increased rearrangements of the cpDNA (Duan et al., 2020; Marechal et al., 2009). But in our hands, after testing six pairs of primers described in the literature, we just obtained either negative or irreproducible results.

The reason why we have not used the approach based on soft-clipping information for the analysis of the mtDNA is because of its abundance in small repeated sequences. In the cpDNA of Arabidopsis Col-0, apart from the large inverted repeat, there are no additional repeated sequences larger than 18 base pairs. These were not retained using our threshold of >20 nuc alignment, and it was easy to discard the reads corresponding to the borders of the large inverted repeats. In contrast, when using the same pipeline for the mtDNA, the very large number of small pairs of repeats results in a very high background of reads with soft-clips that are not real structural variants. When tested, we found 10-fold higher number of soft-clip reads/kb than for the cpDNA, both in WT and mutant libraries.

“Rather, for most transcripts an increased accumulation was observed, as compared to WT plants of the same size (Figure 8A), up to 8-fold in the case of the rps4 transcript.’
An statistical test would be useful to detect significant differences with the wild-type. Also, large increases in expression levels are “observed effects” of radA-induced mtDNA instability. Thus, I disagree with the title of this section.

We agree with the reviewer. The title was changed to: " radA-induced mtDNA instability has no apparent negative effects on the accumulation of mitochondrial transcripts", which more accurately describe the observations. We also conducted a t-test to evaluate significant differences (increase) as compared to WT. Only two were with p<0.01, and asterisks were added in Figure 8A.

Reviewer #2: This is a very interesting manuscript that provides insights into some of the long-standing questions regarding proteins and mechanisms involved in plant mitochondrial DNA recombination and genome maintenance. The distinct role of RadA is shown in this work, and the combination of methods and approaches used lead to data that support the conclusions of the work. I found the manuscript to flow well and the work seems quite complete to me. I do not have any concerns about the experiments or data, and feel that the conclusions made are supported by the work.
The manuscript could be improved for clarity by careful proofreading and editing.

Thank you for your positive appreciation of our manuscript. This new version was thoroughly proofread for typos and awkward sentences.

Two examples:
1. Second paragraph starts 'Defect of the factors...' I think 'defect' is not the correct word to use here and is confusing. Please reword.

Done

2. p. 9 last line, typo, should be 'identified' not 'identifies'

Corrected

Reviewer #3: The authors of this manuscript have characterized an organellar RadA homolog in the model plant Arabidopsis. Using a nice combination of in vitro biochemistry and in vivo studies, they confirm that the gene encodes a true ortholog of the bacterial RadA. While the main function in branch migration has been retained, the work also uncovers some new features that seem to differ from bacterial RadA proteins (e.g., independence of interaction with RecA). The manuscript contains a large amount of experimental data, most of which are of high quality.
I have only a few comments for the authors to consider:
1. The authors suspect the punctate protein localization in chloroplasts to correspond to nucleoids, and used fluorescent protein-tagged PEND to substantiate this conclusion. Whether or not (overexpressed!) PEND::dsRED is a reliable nucleoid marker is somewhat questionable. It would have been more convincing had the authors conducted counter-staining with DAPI, which is generally accepted as a more reliable method of identifying chloroplast nucleoids.

Plastid nucleoids can be visualized after staining with various DNA-specific fluorochromes such DAPI, but by this method the tissue or cell must be fixed with glutaraldehyde or formaldehyde prior to staining. And the cell nuclei emit very bright fluorescence when stained with DAPI, which interferes with the observation of plastid nucleoids. In our hands and in Arabidopsis leaves it is not trivial. That is why we find PEND:GFP or PEND:RFP useful in visualizing plastid nucleoids within intact plant tissues, without need of fixation. In Tarasawa and Sato ([doi.org/10.1093/pcp/pci070](https://doi.org/10.1093/pcp/pci070)) it was demonstrated that in pea protoplasts the PEND:GFP fluorescence coincided with the DAPI counterstaining, consistent with PEND:GFP localizing in chloroplast nucleoids. In Melonek et al. Plant Cell 2012 (doi/10.1105/tpc.112.099721) PEND:dsRED was already used as marker of nucleoids. As in Malonek et al. we also found it to be a bright, low background marker, at least by transient expression in leaves of *Nicotiana benthamiana* following biolistic transformation.

2. For the hemicomplementation experiments, the authors used the RBCS TP to target the protein exclusively to chloroplasts. However, the RBCS TP has been shown to exhibit some leakiness in that it causes (low-level) mistargeting to mitochondria. See, e.g.: Tabatabaei I, et al. (2019) A highly efficient sulfadiazine selection system for the generation of transgenic plants and algae. Plant Biotechnol J 17: 638-649. This problem should be acknowledged and discussed.

Thank you for the information. We cited the reference and included a sentence explaining the problem. But apparently the possible leakage into mitochondria didn't affect our experiments, since the plants transformed with the RBCS:RADA construction showed little or no complementation of the mutant phenotype. We used the endogenous *RADA* promoter, while in Tabatabaei et al. the leaking into mitochondria was probably because of overexpression under 35S promoter.

Minor points:
- Introduction, 2nd paragraph: The first sentence (‘Defect of the factors…’) seems to require some rewording.

Indeed. We thank the reviewer for spotting the error. The sentence was corrected.

- Introduction, last sentence: I don’t think that retrograde signaling ‘mobilizes genes’. I suspect the authors mean ‘activates genes’?

The sentence was corrected

- p. 9: The statement on enlarged mitochondria and the connection of this ultrastructural phenotype to mitochondrial dysfunction and/or impaired gene expression should be referenced and discussed in the context of the existing literature on mitochondrial mutants displaying similar phenotypes (e.g., Zhou W, et al. (2015) Multiple RNA processing defects and impaired chloroplast function in plants deficient in the organellar protein-only RNase P enzyme. PLoS One 10: e0120533).

Thank you for the suggested reference. We cited, together with the paper by Kim et al. 2021, that also reports enlarged mitochondria in *shot1* mutants apparently deficient in the organization of mitochondrial nucleoides.

- The manuscript should be carefully proofread for spelling and grammar (e.g., p.10 ‘Hoever, these values…)’; p.11 ‘…maintenance ant that the loss…’, etc.).

Thank you for spotting those errors. This new version of the manuscript was thoroughly proofread for typos and awkward sentences.
